# Preparing a liquid crystalline dispersion of carbon nanotubes with high aspect ratio

**DOI:** 10.3762/bjoc.20.7

**Published:** 2024-01-11

**Authors:** Keiko Kojima, Nodoka Kosugi, Hirokuni Jintoku, Kazufumi Kobashi, Toshiya Okazaki

**Affiliations:** 1 Nano Carbon Device Research Center, National Institute of Advanced Industrial Science and Technology (AIST), 1-1-1 Higashi, Tsukuba 305-8565, Japan; 2 Department of Chemistry, University of Tsukuba, 1-1-1 Tennodai, Tsukuba 305-8571, Japan

**Keywords:** aqueous dispersion, bar-coating, carbon nanotube, coffee-ring effect, liquid crystal

## Abstract

We successfully prepared a surfactant-assisted carbon nanotube (CNT) liquid crystal (LC) dispersion with double-walled CNTs (DWCNTs) having a high aspect ratio (≈1378). Compared to dispersions of single-walled CNTs (SWCNTs) with lower aspect ratio, the transition concentrations from isotropic phase to biphasic state, and from biphasic state to nematic phase are lowered, which is consistent with the predictions of the Onsager theory. An aligned DWCNT film was prepared from the DWCNT dispersion by a simple bar-coating method. Regardless of the higher aspect ratio, the order parameter obtained from the film is comparable to that from SWCNTs with lower aspect ratios. This finding implies that precise control of the film formation process, including a proper selection of substrate and deposition/drying steps, is crucial to maximize the CNT-LC utilization.

## Introduction

Carbon nanotubes (CNTs) are cylindrical carbon structures with high electrical conductivity and tensile strength in the direction of the tube, anticipated as durable and highly conductive fibers. However, the properties of produced fibers are often inferior to the inherent properties of CNTs. For instance, the wet-spinning method has resulted in CNT fibers with electrical conductivity up to 109,000 S/cm [1], which is about 1/4 that of Al, while individual single-walled CNTs (SWCNTs) have been estimated to have a conductivity of 900,000 S/cm [2]. The electrical conductivity of CNT fibers is primarily determined by the density of the fibers and the length of their constituent CNTs [3–4]. Therefore, enhancing CNT alignment is crucial for producing CNT fibers to endow inherent CNT properties, as greater alignment leads to higher density and consequently improved electrical conductivity.

One effective technique for producing high-performance wet-spun CNT fibers is to utilize liquid crystal (LC) CNT aqueous dispersions as spinning dopes [1,5–10]. The CNT LCs are formed by condensing the dispersed rod-like CNTs, either isolated or bundled [5,7,9–23]. In previous studies, we prepared surfactant-stabilized aqueous dispersions of SWCNT LC and evaluated their dispersion states using the differential centrifugal sedimentation (DCS) method [22–23]. The transition concentrations from the isotropic phase to the biphasic state, and from the biphasic state to the nematic phase were inversely proportional to the aspect ratio (*L*/*D*) of the SWCNT, following the same trend as the Onsager theory [7,9,11,15,20,23–24]. By using dispersions with higher SWCNT aspect ratio, a film of SWCNTs with a higher order parameter was prepared by the bar-coating method [23]. Therefore, it is essential to prepare CNT LCs that contain rod-like CNTs with a high *L*/*D* to fabricate well-ordered CNT materials.

Here, stable double-walled CNT (DWCNT) LC dispersions with a higher *L*/*D* were successfully prepared utilizing highly crystalline DWCNTs. Indeed, the transition concentrations from the isotropic phase to the biphasic coexisting state and from the biphasic coexisting state to the nematic phase were found to be lower than those in the previous study. In addition, the shapes of the spindle-shaped nematic LC, or so-called tactoids, in the biphasic state were examined. The transition from homogeneous tactoids to bipolar tactoids occurred at a larger tactoid volume than that previously observed [23]. This can be ascribed to the higher *L*/*D* of the DWCNTs in this study. On the other hand, despite higher *L*/*D*, the CNT orientation in the film prepared from the dispersion was comparable to the previous study. This suggests that further optimization of the deposition process is necessary to create highly oriented CNT films.

## Results and Discussion

To prepare carbon nanotube dispersions with high aspect ratios, we utilized DWCNTs obtained from Shenzhen Nanotech Port Co. Ltd. Supporting Information File 1 provides characterization details. Briefly, the Raman G/D ratio was approximately 37 (Supporting Information File 1, Figure S1d), indicating high crystallinity [25]. The far-infrared spectrum exhibited a plateau in the lower wavenumber region around 12 cm^−1^, suggesting that a significant amount of DWCNTs have effective lengths (CNT channels consisted of some condutive paths affected by defects or kinks) longer than 4 μm (Supporting Information File 1, Figure S1e) [26].

**Figure 1:**
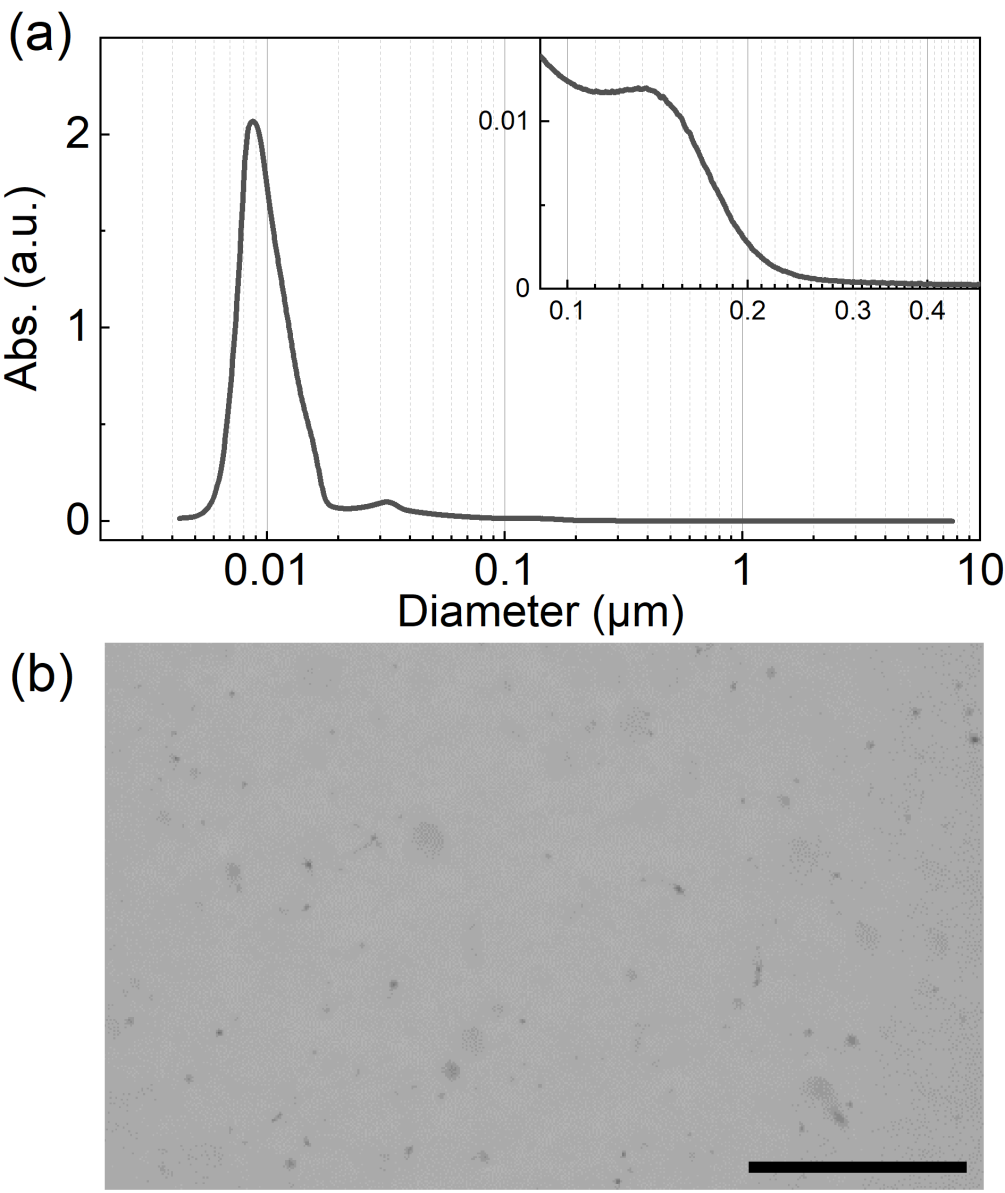
(a) Size distribution of DWCNTs in dispersion by DCS measurements. (b) Optical microscopy image of the DWCNT dispersion. Scale bar is 100 µm.

The DWCNT powder was dispersed with a surfactant of sodium cholate (SC) in water by a two-step process [22–23] (see the Experimental section). The size distribution of the DWCNTs in the dispersion was estimated by DCS method (Figure 1a). The observed intense peak at approximately 9 nm suggests that DWCNTs were mainly dispersed as isolated or bundle forms based on the previous studies [22–23]. Besides, DCS analysis also identified a smaller peak at around 0.1 μm (Figure 1a, inset). The signal can be attributed to DWCNT agglomerates that are 10 μm in size, taking into account the buoyancy resulting from particle porosity [22–23,27–28]. Indeed, the DWCNT agglomerates having corresponding size can be seen in the optical microscope image of the dispersion (Figure 1b).

The shapes of DWCNTs in the dispersion were examined by scanning electron microscopy (SEM) and atomic force microscopy (AFM) (see Supporting Information File 1, Figure S2). In both images, rod-like DWCNTs were observed, while spaghetti-like agglomerates were hardly observed. The average length (*L*) and diameter (*D*) of the DWCNT samples were ≈2.9 μm and ≈2.1 nm, respectively. The *L/D* of the DWCNTs was 1378, which was larger than the value reported in previous research (*L*/*D* = 1088) [23]. Based on the transmission electron microscope (TEM) images, the average diameter of DWCNTs was ≈1.8 nm (Supporting Information File 1, Figure S1a). Since the observed DWCNT diameter based on the SEM images is ≈2.1 nm, the dispersion is likely composed mostly of isolated DWCNTs.

**Figure 2:**
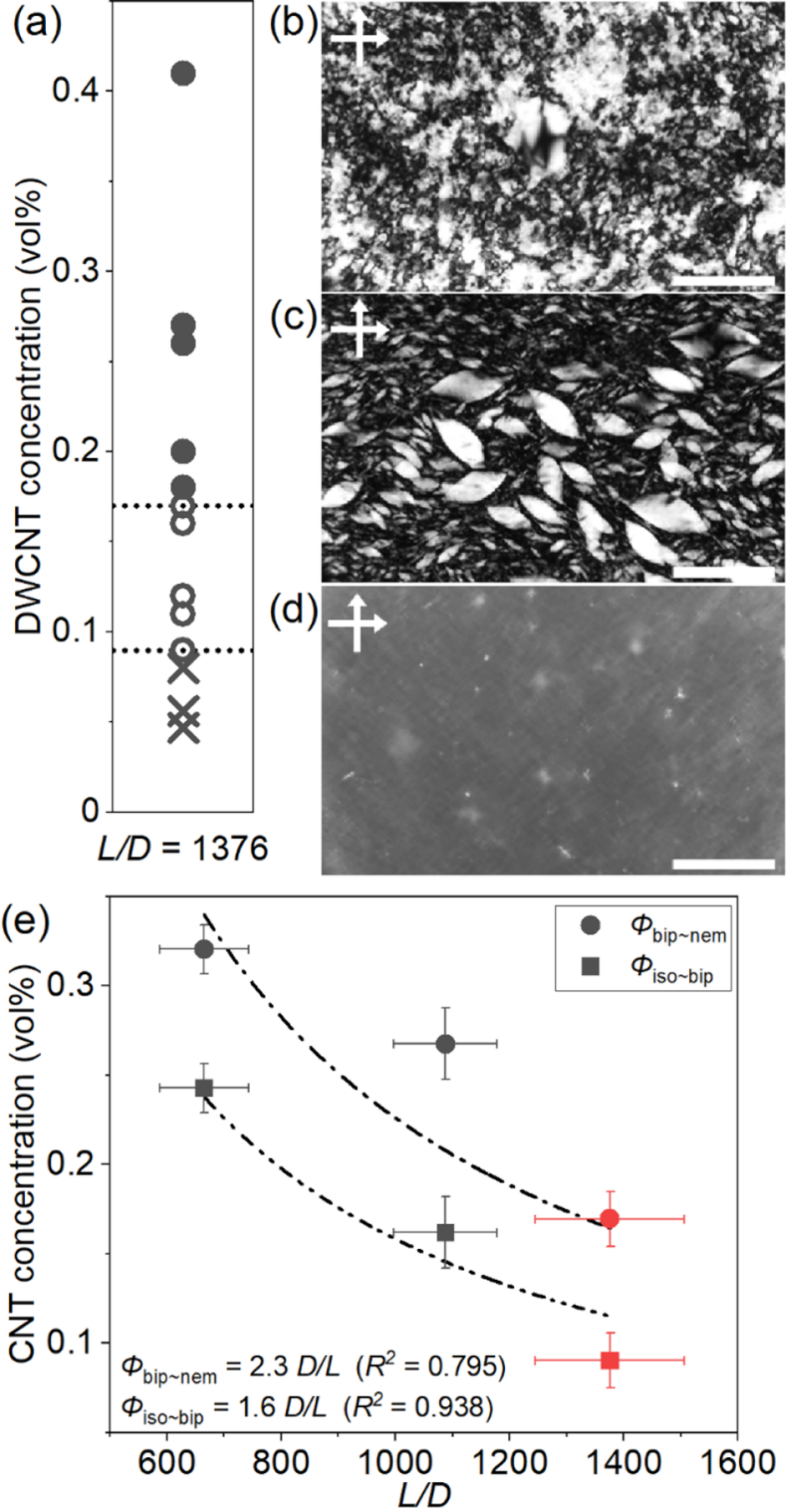
(a) Phase diagram of the DWCNT dispersion. The nematic phase, biphasic state and isotropic phase are marked by filled circle, open circle and cross mark, respectively. (b-d) POM images of the dispersions. DWCNT concentrations are (b) 0.41 vol %, (c) 0.16 vol % and (d) 0.078 vol %. Scale bars are 200 µm. (e) The plot of the phase transition concentration against the CNT aspect ratio *L/D* of three CNT-SC aqueous dispersions [22–23]. Dashed lines indicate the results of curve fitting by inverse function in each phase transition, from isotropic phase to biphasic state and from biphasic state to nematic phase.

To investigate the liquid crystal behavior, the DWCNT dispersion was concentrated by ultra-filtration (Figure 2a). As the concentration of DWCNTs increased, the isotropic phases (Figure 2d, the DWCNT concentration = 0.078 vol %) changed to the nematic phase (Figure 2b, the DWCNT concentration = 0.41 vol %). At intermediate concentrations, biphasic states appeared where the nematic droplets, tactoid, coexisted in the isotropic phase (Figure 2c, the DWCNT concentration = 0.16 vol %) [15,17,22–23,29–30]. The nematic phase shows disturbed textures similar to the previously reported SWCNTs in sodium deoxycholate (SDOC) and sodium taurodeoxycholate (TDOC) aqueous dispersions [23]. The average diameter of DWCNTs in the present study was 2.1 nm, which was almost the same as that of SWCNTs in SDOC and TDOC [23]. Thus, the effect of stiffness of DWCNTs does not seem to affect the domain size of LC very much.

The phase transition concentrations from isotropic phase to biphasic state, ϕ_iso~bip_, and from biphasic state to nematic phase, ϕ_bip~nem_, were estimated by sigmoid curve fitting. The values were found to be 0.090 vol % and 0.17 vol %, respectively, as shown in Supporting Information File 1, Figure S3, which were lower than those previously obtained in the surfactant-assisted SWCNT aqueous dispersions [22–23]. Figure 2e shows the phase transition concentrations obtained in this study (*L*/*D* = 1378, red symbols) together with the previously obtained values for the SWCNT-SC dispersions (*L*/*D* = 1088 and = 665, black symbols) [22–23]. The Onsager theory predicts that the phase transition concentration for rod-like particles with aspect ratio *L*/*D* is ϕ_iso~bip_ = 3.34 *D*/*L* and ϕ_bip~nem_ = 4.49 *D*/*L* [24]. The *L*/*D* dependence of each phase transition concentration shows the inverse proportional relationship.

On the other hand, the surface charge is an important factor for the transition concentrations. In fact, the surfactant dependence can be explained by this effect [23]. The ζ-potential of the DWCNTs was −52.3 ± 0.77 mV (Supporting Information File 1, Figure S4), the repulsive force between the DWCNTs was slightly larger than the previously reported SWCNT-SC dispersions [23]. Although the lower ζ-potential of the DWCNT-SC should lead to higher transition concentration, those of DWCNT-SC were slightly lower than expected based on the fitting curve (Figure 2e). This indicates that the *L*/*D* had a greater impact on the transition concentrations than the ζ-potential.

**Figure 3:**
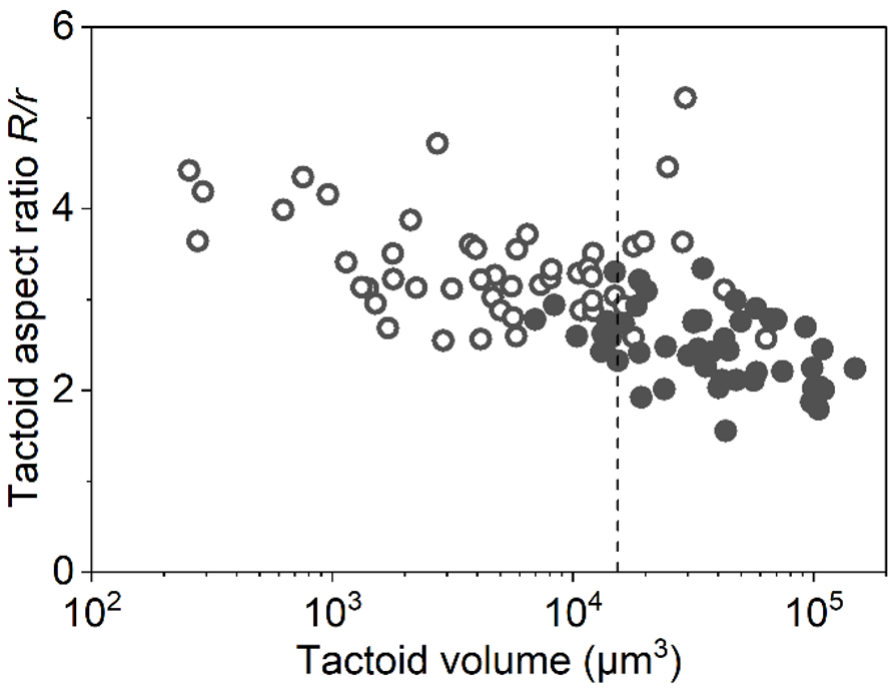
Observed tactoid aspect ratio *R*/*r* as a function of tactoid volume *Rr*^2^. Filled circle and open circle indicate bipolar tactoid and homogenous tactoid respectively. Dashed line denotes the estimated transition volume from homogenous to bipolar tactoids (=15310 μm^3^).

In the biphasic state, the spindle nematic phase droplets, tactoids, were observed (Figure 2c). These were classified into two types depending on the orientation of the DWCNT [22–23,29–30]. If the DWCNTs align parallel to the long axis of the spindle droplet, it is referred to as a homogenous tactoid. Conversely, if the DWCNTs align from one pole to another, it is called a bipolar tactoid. Figure 3 illustrates the aspect ratio of the tactoids (*R*/*r*) as a function of the tactoid volume (*Rr*^2^). We analyzed 100 tactoids in this study. The homogenous tactoids transformed into bipolar tactoids during the volume growth at around *Rr*^2^ = 15310 μm^3^ (dashed line in Figure 3, and Supporting Information File 1, Figure S5). Previous research has shown that the transition volume of tactoids increases with larger *L*/*D* ratios of the SWCNTs [23]. The findings in this study align with these previous results, as the tactoid transition volume and DWCNT aspect ratio were larger compared to previous SWCNT-SC dispersions [23].

**Figure 4:**
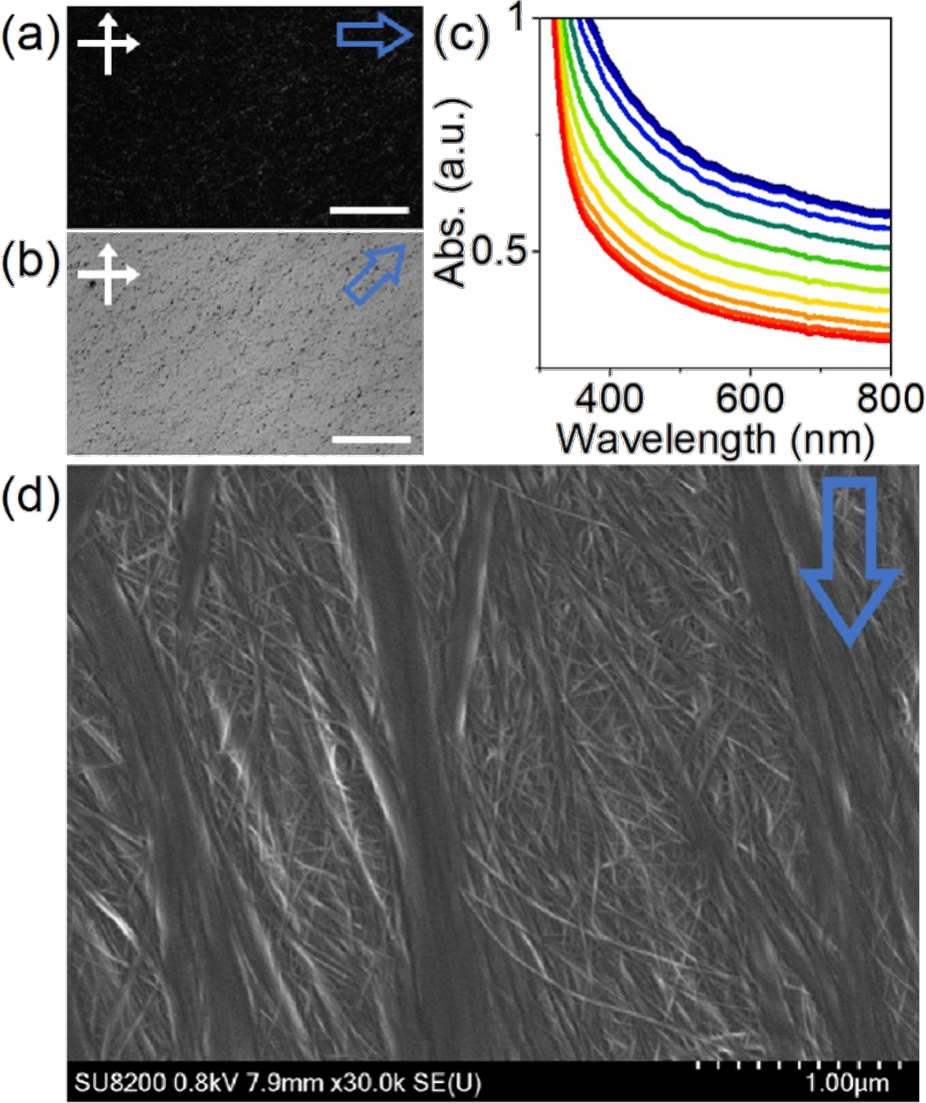
(a, b) POM images of the DWCNT film at (a) 0° and (b) 45° under crossed polarizers (white double arrows). Scale bars are 400 µm. (c) Polarized optical absorption spectra with different angles ranging from 0° (red) to 90° (blue). The sear direction and polarizer are parallel at 0°. (d) SEM image of the DWCNT film. Blue outlined arrows indicate the shear direction of bar-coating.

A thin film was produced utilizing a bar-coating method with DWCNT LC dispersion. The polarizing optical microscopy (POM) images exhibited alignment of the DWCNTs along the shear direction (Figure 4a,b). To estimate the order parameter, the polarized optical absorption measurement was conducted at different sample angles ranging from 0° to 90° at intervals of 10° (Figure 4c). The sample rotation led to gradual changes in absorption intensities, and the average order parameter was calculated to be ≈0.23 from the spectra of 0° (*A*_∥_) and 90° (*A*_⟂_) using Equation 1 [21,23].


[1]

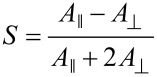



Generally, it is anticipated that rod-shaped particles with larger *L*/*D* will produce a highly aligned film [23,31–32]. However, the value obtained is almost identical to the previous ones with SWCNTs having a smaller *L*/*D* (≈0.22) [23]. Figure 4d shows a typical SEM image of the prepared DWCNT film. One μm wide DWCNT bundles on the film's surface align along the shear direction, while the remaining DWCNTs beneath the thick bundles form mesh-like structures. The shear force exerted by the bar may not sufficiently align the DWCNTs located near the substrate. Thus, optimizing the interaction between the DWCNTs and substrate is necessary to improve the alignment.

**Figure 5:**
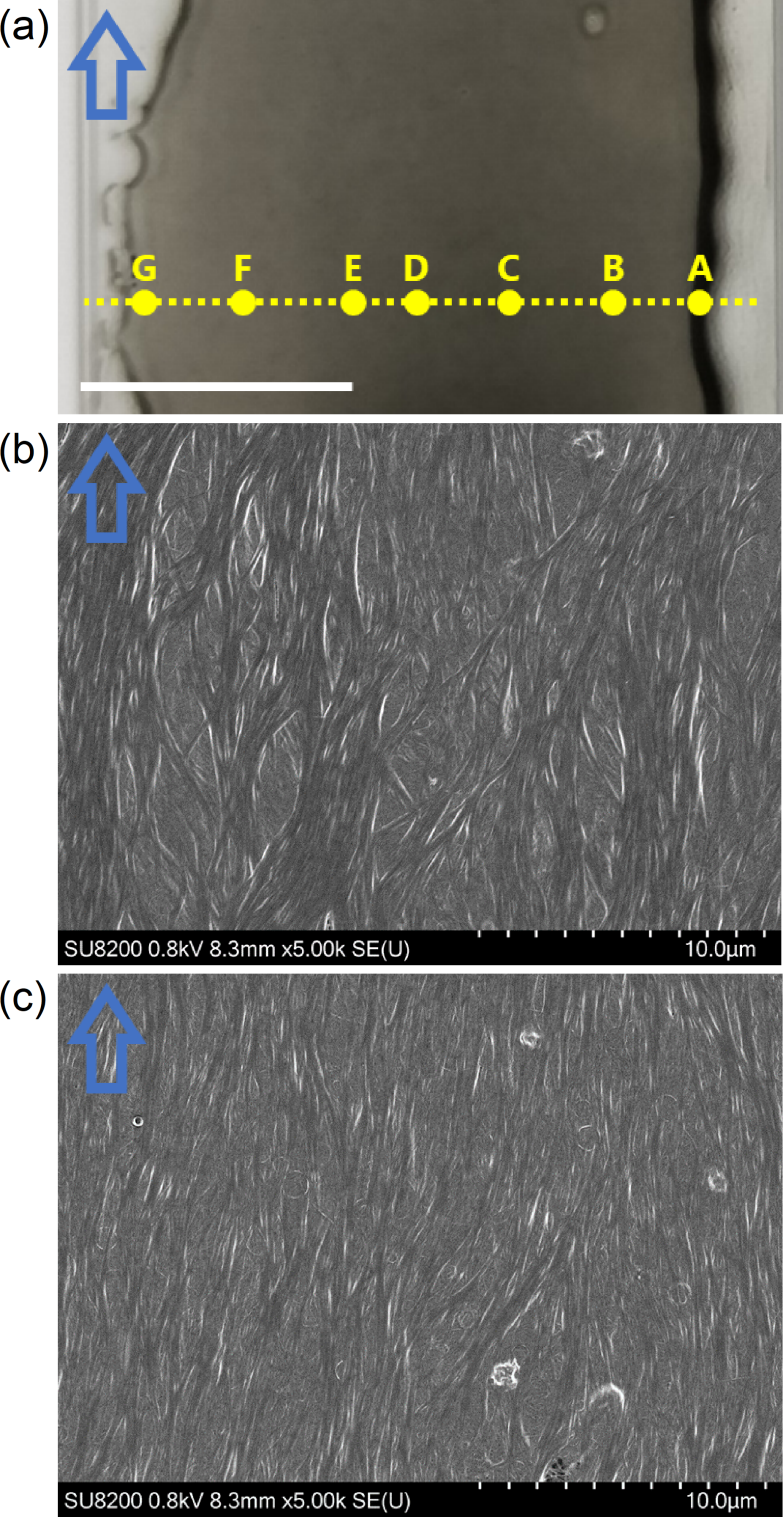
(a) Photograph of the DWCNT film. Thicknesses were measured at 7 spots along the yellow dashed line. Scale bar is 1 cm. SEM image at spot (b) A and (c) D. Blue outlined arrows indicate the shear direction of bar-coating.

To further investigate the low alignment, the film thickness was analyzed by laser confocal microscopy (Figure 5a, Table 1). The average thickness was ≈393 nm, whereas the edge of the film was much thicker (≈999 nm). In earlier studies, the CNTs in the dispersions were observed to flow and accumulate along the droplet's edge due to the coffee ring effect [15,33–34]. As a result, the volume fraction of DWCNTs at the edge increased. Notably, the widths of bundled DWCNTs at the edge were larger than in the inner region (Figure 5b,c). The direction of the bundled DWCNTs near the film surface were parallel to the shear direction in both regions. Due to the randomly oriented DWCNTs beneath the surface, the densification of aligned DWCNTs toward the edge region could account for the similar orientation degree to the previous lower *L*/*D* SWCNT film [23]. To obtain highly aligned CNT films, the condition of drying step [33], the surfactant concentration of the dispersion, the materials of the substrate [34], and the viscosity of the dispersions should be adjusted. For instance, Headrick et al. fabricated highly aligned films by immersing the substrate in a solidifying solution immediately after the SWCNTs were aligned by applying shear stress to the dispersion [35].

**Table 1:** The list of the thickness at each spot in Figure 5a.

Spot	Thickness [nm]

A	999
B	339
C	320
D	343
E	301
F	252
G	200

## Conclusion

We here investigated the LC behavior of the rod-like DWCNTs having a high *L*/*D*. Because the aspect ratio was 1.3 times larger than that of the low *L*/*D* SWCNTs, the phase transition concentrations, ϕ_iso~bip_ and ϕ_bip~nem_ became lower than those reported previously, which is consistent with the Onsager theory. On the other hand we found that the order parameter of the DWCNT film, obtained from the LC dispersion, was nearly identical to that of the film composed of lower *L*/*D* SWCNTs [23]. Further optimization of experimental conditions, including viscosity of the dispersion and substrate, is necessary to fully utilize CNT-LC dispersions.

## Experimental

### Characterization of DWCNT

DWCNTs were purchased from Shenzhen Nanotech Port Co. Ltd. (NTP9112, Product Number DW220817). For the transmission electron microscope observations (Topcon Corporation, EM-002B), DWCNTs were suspended in acetone using a bath-type sonicator and were put onto a copper grid with carbon mesh. To perform thermogravimetric analysis (TGA), a TG/DTA7300 instrument from SII Nano Technology Inc. was utilized. The DWCNT powder was preheated at 180 °C for ten minutes under vacuum and subsequently measured under flowing air (200 mL/min) at a ramping rate of 1 °C/min. The Raman and absorption spectra were obtained from DWCNT thin film on a silicon substrate. The DWCNT thin film was prepared using vacuum filtration. Raman measurements were conducted using a HORIBA T64000 532 nm laser, while far-infrared (FIR) absorption measurements were carried out using a Fourier-transform infrared spectroscopy (Bruker, VERTEX 80v) and a THz time domain spectroscopy (THz-TDS) system (Otsuka Electronics, TR-1000).

### Preparation of the DWCNT dispersion

The dispersion of DWCNTs was prepared using a two-step process in accordance with previous studies [22–23]. Initially, 100 mg of DWCNT powder was pre-dispersed in 100 mL of Wako glycerol with a stirrer (ASONE, HPS-100PD) rotating at 500 rpm for 18 hours. During the stirring, the DWCNTs were defibrated by the shear stress of the high-viscosity glycerol [36]. After suspension in 200 mL of water, the DWCNT-glycerol mixture was filtered through a 1.0 μm pore size polytetrafluoroethylene (PTFE) membrane (Merck, Omnipore 47mm). To ensure complete removal of glycerol from the DWCNTs, we repeated the washing process until 2 L of water had passed through the membrane, resulting in the formation of a DWCNT wet cake. In the second step, the cake was placed in a 50 mL solution of 10 mg/mL sodium cholate (SC) (Wako) and sonicated for 50 minutes using a 500 W tip-type sonicator (Sonics, VCX500) with a titanium alloy tip (TI-6AL-4V). Centrifugation at 4,000*g* for 30 minutes (KOKUSAN, H-36) and then 20,000*g* for 20 minutes (Hitachi-Koki, CS100GXII) effectively removed the large DWCNT agglomerates and impurities remaining in the dispersion. The dispersion states of DWCNTs were assessed using optical microscopy (OLYMPUS, DSX510) and differential centrifugal sedimentation (DCS) analysis (CPS Instruments, CPS 24000UHR (CR-39)). The shape and size of the DWCNTs in dispersion were observed by settling them on a silicon substrate, washed with acetone, and exposed to UV light for 20 minutes using an ozone cleaner (Meiwafosis, PC-450 plus) via dip-coating. The structures of DWCNTs were examined using scanning electron microscopy (SEM) (HITACHI High-Tech, SU8200) and atomic force microscopy (AFM) (OLYMPUS, OLS-4500). Additionally, the ζ-potential of the DWCNTs was determined by measuring the ζ potential analyzer (Malvern Panalytical, ZETASIZER Pro Red label) in a disposable cell (Malvern Panalytical, DTS1070).

### Concentration of the DWCNT dispersion and measurement of liquid crystal phase transitions

The DWCNT dispersion was concentrated by a centrifugal ultrafiltration method (SARTORIUS, VIVASPIN Turbo 15, molecular weight cut-off (MWCO) = 3,000 Da). The value of MWCO was chosen to eliminate only SC and water molecules. The DWCNT dispersion was repeatedly subjected to centrifugation at 1,000*g* for 5 minutes (KOKUSAN H-36) and stirred with a pipette to prevent DWCNT re-aggregation, resulting in the production of the condensed DWCNT dispersion. The dispersions' concentrations were adjusted by adding 10 mg/mL of SC aqueous solution and then estimated from the optical absorption intensity at 750 nm by using a Shimazu UV-3100 spectrometer. Subsequently, each dispersion was enclosed within a Vitro TubeTM 5012-050 glass capillary (cavity size: 0.1 mm × 2 mm × 50 mm) from VITROCOM, and the edges were sealed by UV resin. After the samples were left for one week, the liquid phases were observed using an OLYMPUS BX51 polarized optical microscope (POM).

### Preparation and characterization of DWCNT film

The DWCNT film was prepared from the dispersion with nematic phase by bar-coating method. Initially, the glass slide was washed with acetone and then irradiated with UV light for a duration of 1 hour via an ozone cleaner (Meiwafosis, PC-450 plus). Later, the substrate was coated using a bar coater (OSG SYSTEM PRODUCTS Co., LTD, Select-Roller L60, OSP-10 (bar groove pitch: 0.2 mm, depth: 24 μm)) with the dispersion. The specimen was air-dried until the film was completely dried. The DWCNT orientations of the film were observed by POM (OLYMPUS, BX51) and SEM (HITACHI High-Tech, SU8200). The degree of alignment was determined through analysis of the polarized optical absorption spectra by a Jasco V-770 spectrometer. The film's thicknesses were measured using a laser confocal microscope (OLYMPUS, OLS-4500).

## Supporting Information

File 1 Additional experimental information and figures.
