# Supplementary material for: Preparing a liquid crystalline dispersion of carbon nanotubes with high aspect ratio
Source: Beilstein J Org Chem. 2024 Jan 11;20:52–8. doi: 10.3762/bjoc.20.7 (PMC10790658; doi:10.3762/bjoc.20.7)
Supplement: File 1 — Additional experimental information and figures. [file Beilstein_J_Org_Chem-20-52-s001.pdf]

BEILSTEIN JOURNAL OF ORGANIC CHEMISTRY

Supporting Information

for

Preparing a liquid crystalline dispersion of carbon nanotubes with high aspect ratio

Keiko Kojima, Nodoka Kosugi, Hirokuni Jintoku, Kazufumi Kobashi and Toshiya Okazaki

Beilstein J. Org. Chem. **2024,** *20,* 52–58. doi:10.3762/bjoc.20.7

Additional experimental information and figures

License and Terms: This is a supporting information file under the terms of the Creative Commons Attribution License (https://creativecommons.org/licenses/by/4.0). Please note that the reuse, redistribution and reproduction in particular requires that the author(s) and source are credited and that individual graphics may be subject to special legal provisions.
The license is subject to the *Beilstein Journal of Organic Chemistry* terms and conditions: (https://www.beilstein-journals.org/bjoc/terms)

Characterization of the raw DWCNT powder

To assess the properties of the raw DWCNT powder, we performed TEM, TGA, Raman, and FIR absorption measurements. The TEM image showed an average diameter of ≈1.8 nm (Figure S1 (a)). One hundred CNTs in the raw sample were examined, and the quantitative ratio of DWCNTs to SWCNTs was 55:45. Based on the TGA results, there is very little impurity found in this DWCNT powder (see Figure S1(b)). Additionally, the G/D ratio was ≈37 (Figure S1(d)). Lastly, we determined the effective length of the DWCNTs, which corresponds to the DWCNT length without kinks and defects, by measuring FIR absorption [1]. The effective length of DWCNTs was found to be ≈4200 nm.

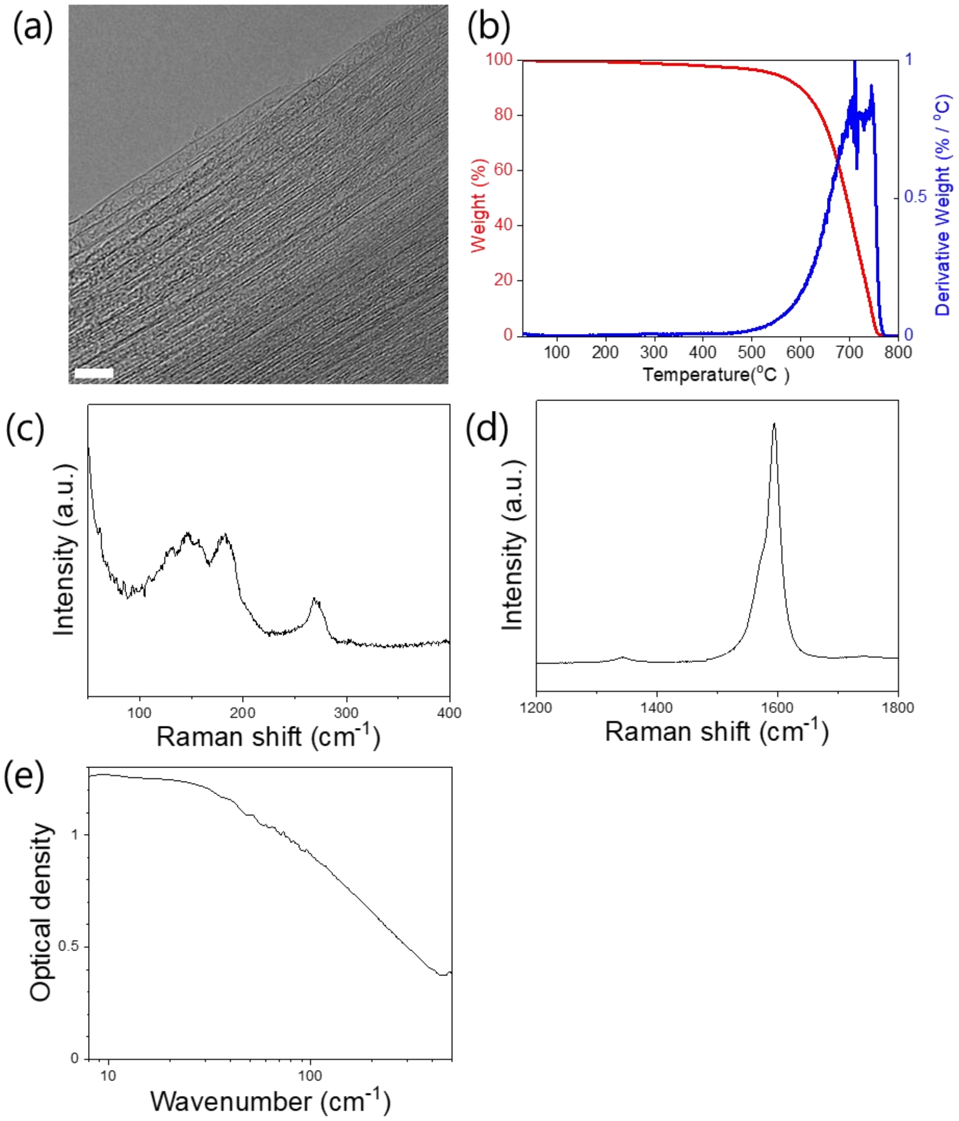

Figure S1: (a) TEM image of DWCNT before dispersing process. The scale bar is 5 nm. (b) TGA results of DWCNT. At the end of analysis, 0.24% of weight were remained. (c,d) Raman spectra of the DWCNT film (532 nm excitation). (c) RBM peaks and (d) G and D bands. (e) Far-infrared absorption spectrum of DWCNT thin film.

Measurements of diameter and length of rod-like DWCNT

The size of the rod-like DWCNTs was observed by scanning electron microscopy (SEM) and atomic force microscopy (AFM). The AFM height profile provided the diameter of the DWCNTs (≈2.10 nm on average), and the SEM image provided its length (≈2.90 μm on average). On the other hand, the longer length is estimated for the raw DWCNT sample (Figure. S1(e)). It is likely that the length of DWCNTs was shortened by the dispersion processes.

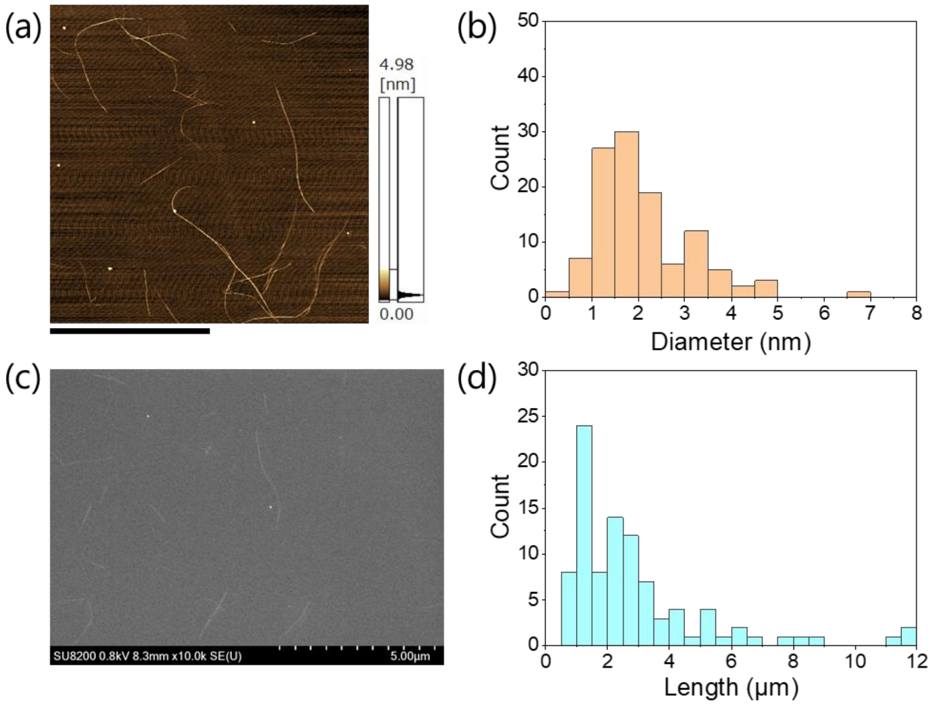

Figure S2: (a) AFM and (c) SEM images of rod-shaped DWCNT in the dispersion. Scale bar is 5 μm. (b) Diameter and (d) length distributions of rod-shaped DWCNT based on AFM and SEM observations, respectively.

Estimation of the phase transition concentrations

To estimate the phase transition concentrations, $\phi_{\text{iso\sim bip}}$ and $\phi_{\text{bip\sim nem}}$, the sigmoid curve fitting was carried out. In Figure S3, the vertical axis represents the isotropic phase, biphasic state, and nematic phase with values of 0, 0.5, and 1, respectively. The fitting result is shown by the red solid line, as determined by Equation S1. The concentration at the points of 0.25 and 0.75 are considered as the transition concentrations from the isotropic phase to the biphasic state, $\phi_{\text{iso\sim bip}}$ ≈0.090 vol%, and from the biphasic state to the nematic phase, $\phi_{\text{bip\sim nem}}$ ≈0.17 vol%, respectively.

$$y = \frac{-1}{1+\ e^{(x-\ x_0)/dx}} \qquad \text{(S1)}$$

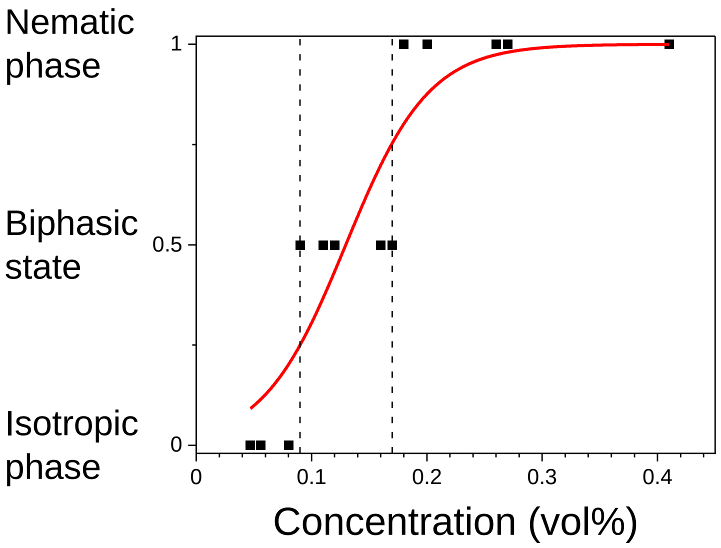

Figure S3: The plot of liquid phases of DWCNT dispersion against DWCNT concentration. Red solid line indicated the result of sigmoid curve fitting.

Surface potential of the rod-like DWCNTs

In order to determine the surface potential of the DWCNTs, six measurements of the ζ potential were carried out. The average of the ζ-potential was −52.3 ± 0.77 mV.

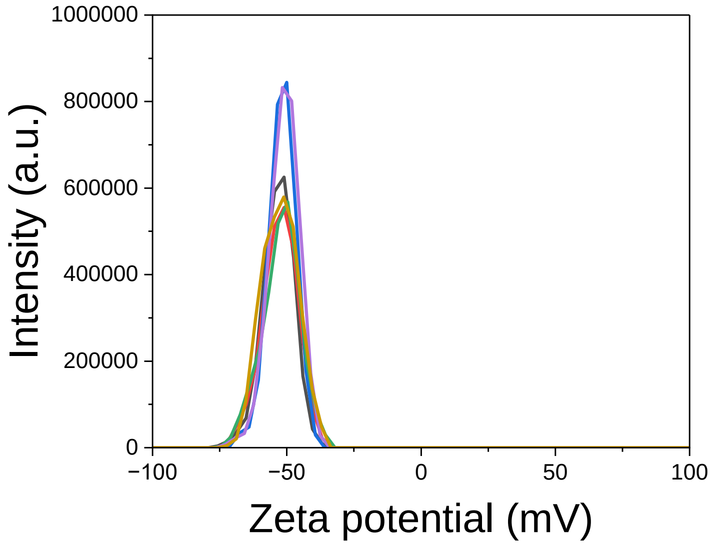

Figure S4: The results of zeta potential measurements of the DWCNT LC sample.

Estimation of the tactoid transition volume

To determine the tactoid transition volume, we conducted a sigmoid curve fitting. In Figure S5, we labeled homogeneous tactoids and bipolar tactoids as 0 and 1 on the vertical axis. The red solid line shows the fitting result of equation S1. At the points of 0.5, the tactoid volume was considered the transition volume from homogenous tactois to bipolar tactoid, $Rr^2 \approx 15{,}310\ \mu m^3$.

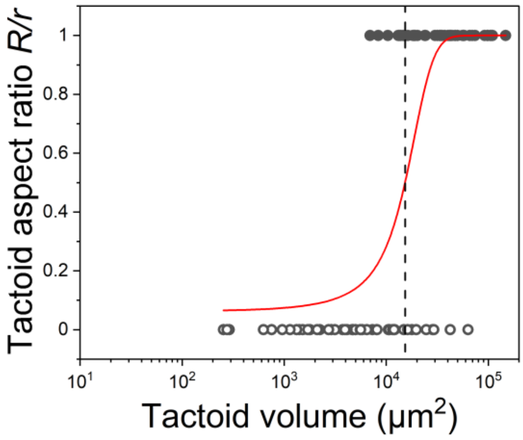

Figure S5: Tactoid aspect ratio *R/r* as a function of tactoid volume Rr^2. Red solid line indicated the result of sigmoid curve fitting.

Reference

1) Morimoto, T.; Joung, S.; Saito, T.; Futaba, D. N.; Hata, K.; Okazaki, T. *ACS Nano* **2014**, *8*, 10, 9897-9904.
